# A High-Fidelity Patient-Derived Organoid Platform Recapitulates the Dynamic Metabolic Landscape of Cisplatin Tolerance in Mesothelioma

**DOI:** 10.3390/cancers18101500

**Published:** 2026-05-07

**Authors:** Zivile Useckaite, Ashleigh J. Hocking, Lauren A. Mortimer, John Salamon, Simon Lee, Yazad Irani, Lucy Franzon, Arya L. Arul, Sarita Prabhakaran, Sonja Klebe

**Affiliations:** 1 Flinders Health and Medical Research Institute, College of Medicine and Public Health, Flinders University, Adelaide, SA 5042, Australia; 2 South Australian Genomics Centre, SAHMRI (South Australian Health and Medical Research Institute), North Terrace, Adelaide, SA 5000, Australia; 3 Department of Surgical Pathology, SA Pathology at Flinders Medical Centre, Adelaide, SA 5042, Australia

**Keywords:** mesothelioma, chemotherapy resistance, therapeutic escape, cancer metabolism

## Abstract

**Background**: Pleural mesothelioma (PM) is characterised by often rapid therapeutic failure and chemotherapy resistance. While terminal resistance is well studied, the initial transition into a drug-tolerant phenotype remains poorly understood. **Methods**: We established patient-derived organoids (PDOs) from malignant pleural effusions to model this transition. Cisplatin-tolerant lines were generated via repeated incremental exposure to cisplatin and compared to time-matched treatment-naive controls using RNA sequencing and Seahorse XFe96 metabolic flux analysis. **Results**: Integrated profiling suggested that the route to tolerance may be influenced by the underlying mutational profile. In this cohort, all BAP1-retained models (including those with KRAS mutations or MTAP loss) adopted an elevated basal metabolic hybrid phenotype, significantly upregulating baseline oxidative phosphorylation and glycolysis to fuel survival mechanisms. Conversely, BAP1-deficient models entered a hypometabolic state of dormancy, characterised by baseline bioenergetic suppression and reduced Ki-67 proliferation. Transcriptomic analysis identified a vesicular transport signature (*SYNGR3*, *VPS52*, *PROM2*) in plastic models, suggesting altered membrane trafficking as a potential survival strategy. **Conclusions**: Our findings demonstrate that mesothelioma therapeutic escape is not a uniform process. Identifying these patient-specific metabolic and transcriptomic trajectories via 3D PDOs provides a hypothesis-generating framework to explore potential avenues for future personalised therapy.

## Simple Summary

Mesothelioma is an aggressive cancer associated with asbestos exposure. Chemotherapy is often rendered ineffective due to the development of resistance, and there is currently no way to predict resistance. We developed an ex vivo 3D organoid (tumour in a dish) model to help identify the mechanism of resistance in mesothelioma. We discovered inter-tumour heterogeneity in mechanisms of chemotherapy resistance. Some tumours may become hyperactive, apparently increasing their energy production to pump out chemotherapy drugs, while others enter a state of dormancy to evade the chemotherapy. These divergent paths may be linked to specific clinical genetic markers, such as expression of the BAP1 protein. Our study highlights the importance of addressing tumour heterogeneity.

## 1. Introduction

Pleural mesothelioma (PM) is an aggressive malignancy characterised by poor prognosis and a susceptibility to rapid therapeutic failure [1]. Despite advancements in immunotherapy, platinum-based chemotherapy remains one of the first-line therapies for mesothelioma, and many patients receive more than one therapy, with the optimal order of different lines of therapy remaining uncertain. Unfortunately, the rapid emergence of cisplatin resistance remains a significant barrier to long-term survival [2].

Current understanding of this resistance is largely derived from models of high-grade, stable resistance; however, these terminal phenotypes often rely on fixed genetic alterations that may obscure the initial, dynamic mechanisms allowing tumour cells to survive first-line therapy [3].

A significant hurdle in the field has been the lack of sophisticated preclinical models that accurately recapitulate the high degree of intra- and inter-patient heterogeneity inherent to mesothelioma [4]. Traditional 2D cell lines fail to preserve the complex genetic architecture and metabolic profiles that drive clinical failure [5]. Furthermore, understanding the initial phase of therapeutic escape is critical. The early cisplatin-tolerant phenotype constitutes a discrete, intermediate biological state that diverges significantly from established, fixed resistance [6,7]. Rather than relying on permanent de novo genomic alterations, this adaptive drug-tolerant state is governed by cellular plasticity, driven primarily by metabolic rewiring and epigenetic remodelling [6,8,9].

This reversible adaptability enables a sub-population of mesothelioma cells to endure therapeutic stress, effectively functioning as a reservoir for recurrent, refractory disease [10]. The transition into this intermediate tolerant state is facilitated by a transcriptomic shift, where specific non-genetic pathways are mobilised to bypass standard cell death pathways [11]. Understanding this transient state requires sophisticated experimental platforms to study cancer’s adaptive potential. The use of patient-derived organoids (PDOs) represents a transformative advance in our ability to model these dynamic processes. Unlike static 2D cultures, PDOs maintain the three-dimensional architecture and cellular diversity of the original tumour, providing a unique co-clinical window into the development of chemoresistance as it occurs in real-time [12]. By preserving the specific genetic drivers of the original malignancy, PDOs allow us to interrogate how underlying genetic compositions dictate the specific tumour trajectory towards cisplatin tolerance. High-fidelity 3D models are essential for identifying novel non-genetic pathways for intervention, such as metabolic dependencies, that are typically lost in traditional models [13].

The emergence of resistance is increasingly recognised as an active and energetic process where metabolic and transcriptomic plasticity converge to overcome a lack of baseline genomic resistance. Metabolic plasticity allows cancer cells to adjust their metabolic phenotypes to adapt to hostile environments [14]. This involves a dramatic increase in “flux”, the total rate at which molecules flow through metabolic pathways to generate ATP [15,16]. By mapping these patient-specific metabolic dependencies, this PDO platform generates hypotheses on how patients might eventually be grouped based on their adaptive capacity. This personalised approach could ultimately inform the discovery of targeted strategies to eradicate the recurrent disease, moving the field towards a more precise and effective therapeutic framework for mesothelioma.

BAP1 and MTAP loss are recognised as key molecular “drivers” in mesothelioma development, with distinct biological consequences that may impact resistance mechanisms, but their clinical applicability in predicting therapy response remains poorly understood [17]. They are already routinely clinically reported and may become useful for clinical treatment stratification.

**Figure 1:**
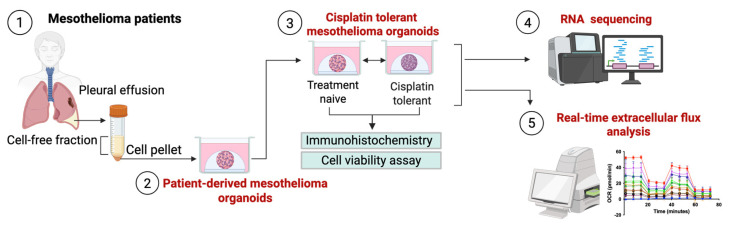
Schematic overview of the experimental workflow. Pleural mesothelioma organoids were established using cells isolated from patient-derived pleural effusion fluids (1); cisplatin-tolerant organoid lines were generated alongside their time-matched, treatment-naive parental controls (2); cell viability, proliferation, and mutation status were confirmed (3); and subsequently, these organoids were collected to perform RNA sequencing (4) and metabolic profiling using real-time extracellular flux analysis using an Agilent Seahorse analyser (5).

In this study, we hypothesise that the route to cisplatin tolerance is not uniform and may be influenced by the tumour’s underlying mutational profile. We leverage PM PDOs to interrogate the critical evolutionary bottleneck where resistance originates. By coupling transcriptomic profiling with real-time metabolic flux analysis, we aim to map non-genetic survival strategies that emerge during this critical evolutionary bottleneck (Figure 1).

## 2. Materials and Methods

### 2.1. Ethics Statement

This study was conducted in accordance with the principles set out in the Declaration of Helsinki. The study was approved with a waiver of informed consent by the Central Adelaide Local Health Network Human Research Ethics Committee (study reference number: 14283), in accordance with the Australian National Statement on Ethical Conduct in Human Research.

### 2.2. Pleural Effusion Collection and Sample Processing

Pleural effusion fluids were collected in excess of diagnostic requirements from people diagnosed with mesothelioma, submitted to the Department of Cytopathology, Flinders Medical Centre, between May 2021 and July 2024, following routine cytopathological screening. All clinical diagnoses were reviewed by two Royal College of Pathologists of Australia (RCPA)-qualified pathologists. All diagnoses were made in line with current (2021) WHO diagnostic recommendations and current guidelines [18]. Pleural effusion fluids were centrifuged for 10 min at 500× *g* at 4 °C. Pleural effusion supernatant was removed, aliquoted, and stored at −80 °C. Cell pellets containing red blood cells were treated with 3 mL of Ammonium–Chloride–Potassium (ACK) Lysing Buffer (Thermo Fisher Scientific, Waltham, MA, USA) for 1 min in a 37 °C bead bath, which was then deactivated with 12 mL of Advanced Dulbecco’s Modified Eagle Medium/Ham’s/F-12 (Gibco) and centrifuged at 400× *g* at 4 °C for 5 min. The subsequent cell pellet was then processed for cell culture.

### 2.3. Patient-Derived Organoid Culture

Patient-derived organoids (PDOs) were established from malignant pleural effusions and characterised as previously described [19]. Organoids were maintained in Advanced DMEM/F12 (Gibco) containing 10% mesothelioma–pleural effusion supernatant. All cultures were confirmed to be mycoplasma negative using a PCR-based Mycoplasma Detection Kit (Applied Biological Materials, Richmond, BC, Canada).

### 2.4. Establishing Cisplatin-Tolerant Organoids

Cisplatin-tolerant organoid lines were established using treatment-naive PDOs at early passages (following establishment, passages 4–5). Baseline dose–response curves were generated for each line using an 8-point cisplatin dilution series to estimate IC50 and PDOs were selected based on their initial sensitivity towards cisplatin. Organoid cultures were treated with increasing concentrations of cisplatin over time, as previously described [20]. Cisplatin treatments were continued until the cultures had reached >3-fold difference in the IC50 value of the untreated control, with the exception of one PDO (PDO2), which developed intrinsic resistance in the treatment-naive pair (IC50 = 1.75). Matched untreated control organoids were maintained in culture simultaneously to account for changes in resistance over time and provide a time-matched treatment-naive pair.

### 2.5. Immunohistochemistry

Organoids were prepared for histology and immunohistochemistry by melting organoid–Matrigel domes in 10 mL of ice-cold 1× PBS for 10 min, followed by centrifugation at 400× *g* for 5 min at 4 °C. Organoid pellets were fixed in 4% formalin solution for 10 min at room temperature. Fixed organoids were resuspended in pre-melted 3% agar and placed in individual plastic meshed cassettes where they were subjected to the following dehydration stages in a Leica PELORIS Rapid Tissue Processor (Leica Biosystems, Nussloch, Germany): 10% formalin (90 min), 85% ethanol (20–60 min, 6 changes), xylene (30–90 min, 3 changes), and embedded in paraffin wax (60–90 min, 3 changes). Immunohistochemistry using diagnostic antibodies was performed on 4 μm thick paraffin sections. Antibody labelling was carried out according to validated clinical procedures approved by the National Association of Testing Authorities (NATA, Sydney, Australia) on the Ventana BenchMark ULTRA Automated IHC/ISH platform (Ventana Medical Systems, Tuscon, AZ, USA). Antigen retrieval was performed using Ventana reagent Cell Conditioning Solution 1 and a Ventana Amplification Kit (760-080) (Ventana Medical Systems, Tuscon, AZ, USA). Antibody-positive control tissue for validation was included on every slide, consistent with clinical diagnostic requirements. Images were taken using a BX53 light microscope (Olympus Corporation, Tokyo, Japan).

### 2.6. Ki67 Proliferation Index

Proliferation rates were quantified using the Ki67 labelling index, defined as the percentage of positively stained nuclei. For each organoid line, at least 100 cells were counted in hotspot areas, using a modular upright microscope (Olympus BX52; Olympus Corporation, Tokyo, Japan) at 40× magnification. Immunohistochemical staining was performed on formalin-fixed, paraffin-embedded organoid sections using the Ventana Ki67 clone 30-9 predilute following a NATA-approved protocol on the Ventana Benchmark Ultra.

### 2.7. Cell Viability and Half-Maximal Inhibitory Concentration Determination

Cisplatin tolerance was quantified by determining the IC50 using the Celltiter-Glo^®^ 3D Cell Viability Assay (Promega, Madison, WI, USA). Briefly, PDO growth rates in response to cisplatin treatment (Accord Healthcare, Durham, UK) were assessed by comparing the IC50 values of cisplatin-treated and treatment-naive passage-matched pairs. Organoids were dissociated using TrypLE Express (Gibco, Grand Island, NY, USA) and plated in 90% Matrigel growth medium (1.8 × 10^5^ cells/mL) in a 48-well flat-bottomed plate precoated with 5 µL of 100% Matrigel (Corning Inc., Corning, NY, USA). Organoids were mechanically dissociated and passed through a 40 µm nylon mesh cell strainer (Corning Inc., Corning, NY, USA) to obtain a single cell suspension. Passaged organoids were cultured for six days in minimal medium (ADVancedDMEM/F12 + 1% glutamax, N2 and EGF) containing 10% autologous–pleural effusion supernatant to allow the organoids to reach approximately 50 µm in diameter. Organoids were then treated with a serial dilution of cisplatin of either 160, 80, 10, 5, 2.5, 1.25, 0.625, 0.15620, 0.078125, or 0 µM (vehicle-only control) and grown for an additional 6 days. Media containing cisplatin and vehicle control were replenished every 2 days. Cell viability was measured on day 12 using the Celltiter-Glo 3D Cell Viability Assay (Promega) according to the manufacturer’s instructions. Luminescence was measured using a Spectramax iD5 multimode microplate reader at 500 milliseconds (Molecular Devices, San Jose, CA, USA). Assays were performed in technical triplicate across separate experiments. The Resistance Index (RI) of organoids treated with cisplatin was determined using Graphpad Prism 9 (GraphPad Software, Boston, MA, USA) using the following formula: RI = IC50 of resistant cells/IC50 of treatment-naive cells.

### 2.8. RNA Isolation from Patient-Derived Organoids

To generate cell pellets for RNA extraction, organoids were harvested from four Matrigel domes. Briefly, 1 mL of ice-cold PBS was added to each well to depolymerise the matrix. The domes were mechanically disrupted via pipetting, and the suspension was pooled and incubated on ice for 10 min to ensure complete solubilisation of the Matrigel. The organoids were pelleted by centrifugation at 400× *g* for 5 min at 4 °C. Following the removal of the supernatant, total RNA was isolated from the cell pellets using the RNeasy Mini Kit (Qiagen, Hilden, Germany) according to the manufacturer’s instructions.

### 2.9. RNA Sequencing

Library preparation was performed using the Tecan Universal Plus Total RNA-Seq kit, according to the manufacturer’s instructions. The library was sequenced on one lane of an Illumina NovaSeq X Plus (2 × 151 bp) with 2% PhiX spike-in. This yielded an average of 192 M pass-filter reads per sample. Following demultiplexing, data were processed using nf-core/rnaseq (v3.21.0) of the nf-core collection of workflows [21], utilising reproducible software environments from the Bioconda [22] and Biocontainers projects [23]. Briefly, reads were aligned to GRCh38.p14 (NCBI Annotation Release 110) using STAR (v2.7.11b), with Salmon (v.10.3) for transcript quantification, and summarised to gene count tables with tximport (v1.20.1). The pipeline was executed with Nextflow v24.10.3 [24].

### 2.10. Metabolic Flux Analysis

Mitochondrial respiration and glycolysis were simultaneously measured in cisplatin-tolerant and time-matched treatment-naive pairs, using an Agilent Seahorse XFe96 Extracellular Flux Analyzer. Cisplatin-tolerant and treatment-naive pairs were grown in 50 µL domes of 90% Matrigel (Corning) in a 24-well flat-bottomed plate precoated with 10 µL of 100% Matrigel (Corning) for four to six days to allow the organoids to reach approximately 50–100 µm in diameter. Organoid–Matrigel domes were melted in 10 mL of ice-cold 1x PBS for 20 min, followed by centrifugation at 400× *g* for 5 min at 4 °C. Organoids were seeded into a 96-well microplate as 2 μL Matrigel domes in 4–5 technical replicates (approximately 20 PDOs per microwell, making sure that the domes are placed in the centre of the well and sides do not touch the sides on the microwell. Organoids were seeded for the experiment 18 h prior to the assay. Overnight recovery was required to ensure adherence and to mitigate organoid-dissociation-induced metabolic stress and to return to the true basal metabolic rate.

The assay was run as per the manufacturer’s recommendation. Briefly, one day prior to the assay, the sensor cartridge was hydrated by adding 200 mL of sterile tissue culture grade water (Baxter International Inc., Deerfield, IL, USA) to each well of the utility plate and placing the cartridge in a non-CO_2_ incubator at 37 °C overnight. On the day of the assay, the water was removed and replaced with 200 mL of prewarmed calibrant and incubated at 37 °C for 60 min prior to use.

Organoid growth media was removed from all PDOs and replaced with 200 mL of prewarmed to 37 °C XF DMEM medium (pH 7.4, supplemented with 10 mM glucose, 1 mM pyruvate, and 2 mM L-glutamine) and placed in a non-CO_2_ incubator at 37 °C for 1 h. An assay was run to measure basal respiration only. Normalisation was achieved through manual counting of the number and size of organoids in each well using Fiji ImageJ, version 2.14.0. Multi-point and freehand tools, respectively.

### 2.11. Data Analysis

Statistical analyses were performed using GraphPad Prism software (Version 10.0; GraphPad Software, Boston, MA, USA). Differences between passage-matched treatment-naive and cisplatin-tolerant organoid lines were evaluated using a paired, two-tailed Student’s *t*-test. Data are presented as mean ± standard deviation (SD), with a *p*-value < 0.05 considered statistically significant. Given the proof-of-concept sample size (*n* = 4), we acknowledge that the assumptions of normality required for parametric testing cannot be definitively confirmed; therefore, statistical comparisons between groups are interpreted as exploratory.

Differentially expressed genes were identified from Salmon-quantified counts using DESeq2 (v1.34.0) [25], implemented via nf-core/differential abundance v1.6.0dev. Paired sample identity was specified as a blocking variable in the contrast design. Benjamini–Hochberg-adjusted *p*-values were used to correct for multiple testing. Variance-stabilised counts from DESeq2 were used to perform principal component analysis (PCA) on the top 500 most highly variable genes using the shinyngs R package (v2.2.4). Heatmap generation and hierarchical clustering were performed using the pheatmap R package (v.1.0.13).

## 3. Results

### 3.1. Morphological and Histological Characterisation of Mesothelioma Organoid Models

**Figure 2:**
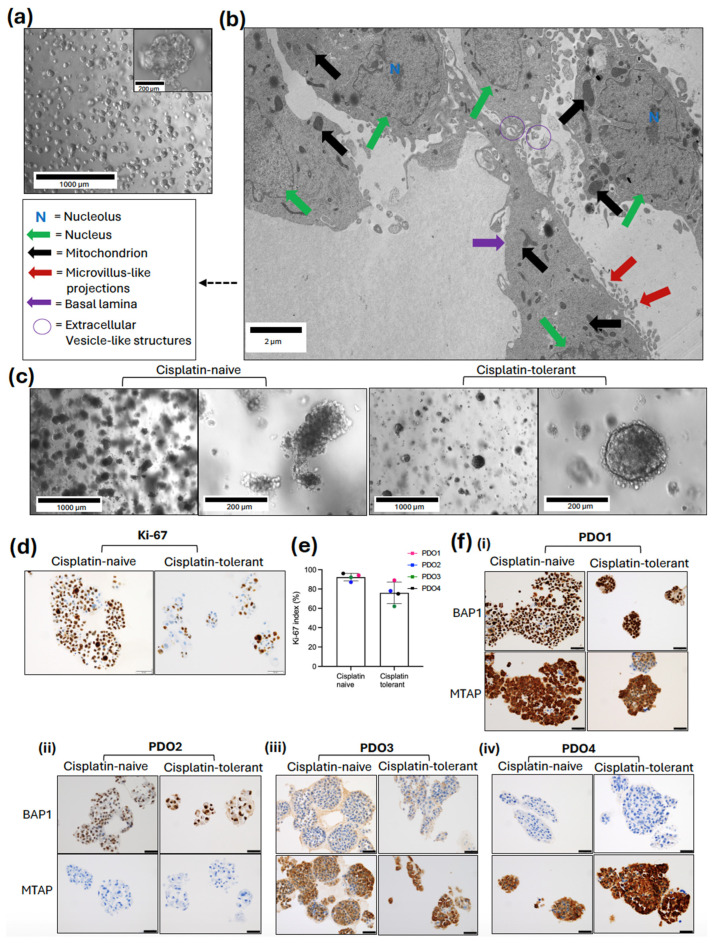
Morphological and histological characterisation of cisplatin-tolerant mesothelioma organoids. (**a**) Representative brightfield microscopy showing successful organoid establishment at early passage (*p*), *p* = 5; (**b**) transmission electron microscopy (TEM) image confirming the retention of key ultrastructural features; (**c**) comparative morphology of treatment-naive and cisplatin-tolerant matched pairs after long-term culture (*p* = 23); (**d**–**f**) immunohistochemical profiling (IHC): (**d**) representative IHC overview of Ki-67; (**e**) Ki-67 staining quantifying the tumour proliferation index in naive versus tolerant pairs. Data presented as mean ± SD; (**f**) assessment of diagnostic biomarkers BAP1 and MTAP (PDO1 (**i**), PDO2 (**ii**), PDO3 (**iii**), PDO4 IV (**iv**)). All PDO models demonstrated complete concordance with the original diagnostic pleural fluid, confirming that the acquisition of drug tolerance occurs independently of alterations in BAP1 or MTAP status. Positive expression is indicated by brown nuclear (BAP1) or cytoplasmic (MTAP) staining. Scale bar = 50 μm.

Brightfield microscopy confirmed the successful establishment of mesothelioma PDOs by passage 5 (*p* = 5) (Figure 2a). The PDOs appeared as three-dimensional multicellular clusters with distinct borders, effectively recapitulating the cellular diversity and architecture. The 3D organoids exhibited a predominantly solid, oval morphology consistent with the epithelioid subtype. By passage 5, the models demonstrated stable growth and morphological uniformity.

Sub-cellular architecture of the established models was evaluated by means of transmission electron microscopy (TEM) at 1900× magnification (Figure 2b). The electron microscopy analysis confirmed the high-fidelity recapitulation of epithelioid mesothelioma, characterised by elongated microvilli-like structures. The cells exhibited large, irregular nuclei with prominent, electron-dense nucleoli, and a high density of mitochondria with intact cristae and extracellular vesicle (EV)-like structures within the intercellular spaces and budding from the plasma membrane.

To evaluate the phenotypic impact of acquired resistance, brightfield microscopy was used to compare the morphological characteristics of time-matched (*p* = 23) cisplatin-naive and cisplatin-tolerant PDOs (Figure 2c). Morphological characterisation revealed distinct phenotypic differences between the two cohorts. While both treatment-naive and cisplatin-tolerant PDOs maintained 3D architecture, the cisplatin-tolerant models displayed a more compact and spherical morphology and dense cellular packing. These structural deviations correlated with altered growth: treatment-naive PDOs exhibited higher proliferative capacity, requiring passaging every 10–12 days, compared to the 14-day cycle required for cisplatin-tolerant pairs. To quantify the proliferative capacity of the models, immunohistochemistry (IHC) analysis was performed to evaluate the expression of the proliferation marker Ki-67. Distinct differences in proliferation rates were observed between the treatment-naive and cisplatin-tolerant pairs. Treatment-naive organoids exhibited a larger proportion of cells with positive nuclear Ki-67 expression compared to the tolerant cells (Figure 2d).

Quantitative assessment indicated a trend towards a higher Ki-67 proliferation index in the treatment-naive pairs relative to their cisplatin-tolerant pairs, though this reduction did not reach statistical significance (*p* = 0.065) (Figure 2e). The mean cell proliferation (±SD) was 92.25 ± 3.86 in cisplatin-tolerant PDOs and 76.00 ± 11.11 in treatment-naive PDOs.

To validate the histological fidelity of our models, we assessed BAP1 and MTAP expression via IHC (Figure 2f). BAP1 and MTAP are clinically relevant histological markers that are routinely used for the diagnosis of malignant versus reactive mesothelial lesions and that may have prognostic and predictive implications [17,18].

The PDOs exhibited complete concordance with the labelling profiles of the corresponding diagnostic pleural fluid and biopsy specimens (where applicable). Specifically, PDO1 retained both markers (Figure 2f(i)), PDO2 exhibited MTAP loss (Figure 2f(ii), while PDO3 and PDO4 displayed BAP1 loss with intact MTAP expression (Figure 2f(iii,iv)). This mutational profile remained stable across all corresponding cisplatin-tolerant sublines, confirming that the acquisition of drug tolerance was not driven by alterations in these foundational diagnostic biomarkers.

### 3.2. Distinct Molecular Landscapes of Mesothelioma PDO Models

**Figure 3:**
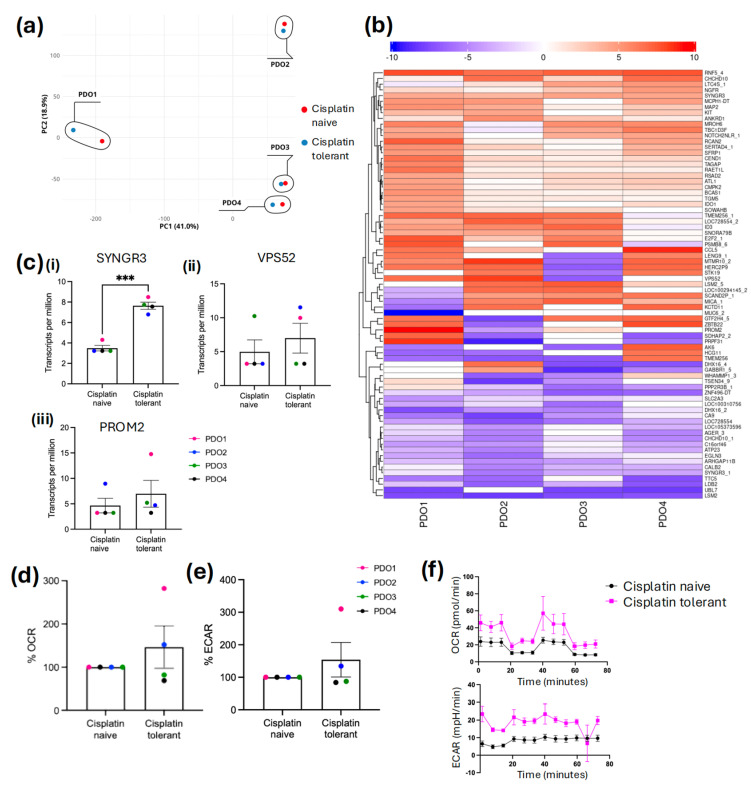
Integrated transcriptomic and bioenergetic profiling reveals distinct mechanisms of cisplatin tolerance. (**a**–**c**) Transcriptomic landscape of resistance: (**a**) Principal component analysis (PCA) demonstrating global divergence between parental and cisplatin-tolerant pairs (transcripts per million); (**b**) heatmap displaying the top 75 differentially expressed genes (Log2FC of DESeq2 normalised counts); (**c**) the expression signature reveals the induction of a vesicular transport, characterised by the significant upregulation of *VPS52* (**ii**), *PROM2* (**iii**), and *SYNGR3* (**i**) (mean ± SEM, *** *p* < 0.001); (**d**–**f**) real-time metabolic flux analysis. Assessment of (**d**) Relative Oxygen Consumption Rate (OCR) and (**e**) Extracellular Acidification Rate (ECAR) using the Seahorse XFe96 analyser; (**f**) representative mitochondrial stress test trace.

Principal component analysis (PCA) was performed to evaluate the global biological variation across four mesothelioma PDO models (Figure 3a). The first two principal components account for 59.9% of the total variance. The four PDO models were segregated into distinct regions of the PCA space, reflecting significant inter-patient heterogeneity. PDO3 and PDO4 clustered closely along the PC1 axis (x~100–150). This shared spatial proximity correlates with their genetic signature (BAP1 loss with MTAP retained).

Evidence suggests that BAP1 alterations are a promising candidate for disease stratification in pleural mesothelioma. Specifically, patients harboring germline BAP1 mutations often demonstrate extended overall survival [17], but the reasons remain poorly understood. BAP1 loss may improve chemotherapy response, as shown in a large-scale study that identified BAP1 loss detected via immunohistochemistry as a predictor of chemotherapy response [26]. Patients with BAP1-deficient tumours showed significantly improved survival when treated with first-line platinum and pemetrexed compared to those who received no active treatment. Conversely, patients with retained BAP1 expression derived minimal benefit from chemotherapy, showing median survival rates similar to those of untreated patients [26]. However, the BAP1 germline status for these patients was unknown. Little is known regarding the predictive value of BAP1 status in the second-line therapy setting. In contrast, the prognostic value of BAP1 in surgical and cytology specimens remains unclear, with some studies showing significantly poorer survival, whereas others show improved survival [17,27].

In this study, PDO1 (BAP1 and MTAP retained, KRAS mutation) was markedly distinct from all other models, localising at the negative end of the PC1 axis (x~−350), suggesting a fundamentally different molecular profile compared to the BAP1-deficient models. A significant role for KRAS in conferring platinum resistance is recognised in NSMC and other solid cancers, but, whilst suspected, has not been shown in mesothelioma [28]. The features of PDO2 (BAP1 retained, MTAP loss, acral lentiginous melanoma, patient diagnosed with prostate cancer localised at the positive end of the PC2 axis (y~200)) distinguished it from the other three models. The patient did not fulfil the current criteria to qualify for germline BAP1 testing [29].

For PDO2, PDO3, and PDO4, the cisplatin-tolerant replicates clustered tightly with their passage-matched treatment-naive controls, indicating that the underlying patient-specific molecular signature remained dominant over the changes induced by cisplatin exposure. Conversely, PDO1 showed greater separation between its cisplatin-tolerant and treatment-naive pairs compared to the other models, suggesting that cisplatin tolerance in this specific genetic background (BAP1/MTAP retained) may involve a more substantial global molecular shift.

Principal component analysis demonstrated biological variations across the four PDO models; however, no significant differences were observed in the initial cisplatin concentrations required to induce a cisplatin-tolerant state (Table S1). To generate cisplatin tolerance, organoids were exposed to cisplatin until they achieved a >3-fold increase in IC50 relative to their passage-matched untreated controls. Notably, PDO3 and PDO4, both harboring IHC-confirmed BAP1 loss, exhibited the highest resistance indices (defined as the fold-change in IC50 between T0 and the cisplatin-tolerant state; Table S1). While germline BAP1 status remained undetermined due to clinical testing constraints, the BAP1-deficient phenotype in these models correlated with the most robust transition to a chemo-resistant state.

To understand the molecular drivers of therapeutic escape, we performed comparative RNA sequencing of cisplatin-tolerant PDOs versus their treatment-naive pairs. This analysis identified 241 differentially expressed genes associated with the tolerant state (Table S2). Hierarchical clustering of the top 75 significantly altered genes revealed a distinct transcriptional shift away from conventional apoptosis pathways (Figure 3b). Due to profound inter-patient heterogeneity observed across the four distinct genetic backgrounds, global pathway enrichment analysis yielded highly divergent network signatures. To identify universal drivers of tolerance, the focus of analysis was on the most strictly conserved, highly significant individual transcripts shared across the divergent models. Notably, we observed the enrichment of a putative vesicular transport signature involving VPS52 and PROM2 Figure 3c(ii) and Figure 3c(iii) respectively, genes that have been previously associated with the non-traditional compartmentalisation of chemotherapeutic agents [30,31], though their distinct role in cisplatin sequestration in this study requires functional validation. The identification of significantly elevated SYNGR3 expression in cisplatin-tolerant PDOs (Figure 3c(i) pinpoints an unexpected mechanism of mesothelioma survival, with some clinical cohorts associating high SYNGR3 expression with a more favourable prognosis in treatment-naive patients, while its upregulation in our post-treatment models suggests a potential functional pivot towards a cisplatin-tolerant phenotype [32,33].

Our data show that SYNGR3 was significantly upregulated in all four cisplatin-tolerant PDOs (*p* < 0.001), and in contrast, VPS52 and PROM2 displayed inter-patient heterogeneity. VPS52 was upregulated in PDO1 and PDO2 but downregulated in PDO3; similarly, PROM2 was upregulated in PDO1 and PDO3 but downregulated in PDO2. PDO4 showed no significant changes in either VPS52 or PROM2. These correlative data suggest that vesicular trafficking may be used as a survival strategy. While the specific molecular machinery employed may vary between patients, the consistent upregulation of SYNGR3 highlights it as a promising candidate for future functional validation.

Metabolic plasticity is frequently associated with therapeutic escape; therefore, bioenergetic profiling was used in this study to characterise the cisplatin-tolerant state [34]. By comparing the real-time metabolic flux of tolerant organoids against their passage-matched treatment-naive pairs, this assay aimed to understand drug-induced adaptive rewiring and to identify specific metabolic dependencies acquired during the evolution of tolerance.

To quantify bioenergetic shifts, the oxygen consumption rate (OCR) and extracellular acidification rate (ECAR) values of treatment-naive organoids were set to a baseline of 100%, with measurements from cisplatin-tolerant pairs expressed as a percentage relative to these controls (Figure 3d,e). In the BAP1/MTAP-retained line (PDO1), the tolerant phenotype was characterised by upregulation of baseline cellular metabolism, exhibiting an upregulation in both mitochondrial and glycolytic activities, reaching 280% (OCR) and 310% (ECAR) of the treatment-naive pair.

Similarly to PDO1, PDO2 analysis revealed that cisplatin-tolerant organoids exhibited a hybrid metabolic phenotype characterised by the simultaneous upregulation of both basal mitochondrial respiration, with a 52% and 34% increase in basal ECAR, compared to treatment-naive controls (Figure 3d,e).

In contrast to the elevated basal metabolic phenotype observed in BAP1-retained models, the BAP1-loss/MTAP-retained PDO3 and PDO4 displayed a distinct downregulation of bioenergetic activity upon acquiring cisplatin tolerance. Both organoid lines exhibited a reduction in mitochondrial respiration, with basal OCR decreasing by 12% in PDO3 and 31% in PDO4 compared to treatment-naive pairs (Figure 3d,e). A simultaneous suppression of glycolytic flux was also observed, with ECAR decreasing by 12% and 14%, respectively. This concurrent reduction in oxidative and glycolytic parameters classifies these organoids into a hypometabolic energy phenotype, suggesting a shift towards metabolic dormancy rather than plasticity.

Bioenergetic profiling analyses were restricted to baseline OCR and ECAR to ensure data fidelity. In 3D culture systems, the Matrigel dome acted as a physical diffusion barrier, introducing a significant lag in the penetrance of mitochondrial uncouplers (especially carbonyl cyanide-p-trifluoromethoxyphenylhydrazone (FCCP)), as this assay is primarily optimised for 2D monolayers. This diffusion latency prevented the establishment of a stable maximal respiration plateau within standard assay intervals, leading to an underestimation of maximal capacity (Figure 3f). Therefore, basal metabolic measurements were prioritised to accurately reflect the real-time bioenergetic state of the PDOs. Consequently, claims regarding metabolic plasticity are limited to baseline shifts. Future studies will use Cell-Tak adherence to bypass the Matrigel diffusion barrier, enabling the complete quantification of maximal respiration and spare respiratory capacity.

## 4. Discussion

In this study, we leveraged PDOs to interrogate the critical cisplatin tolerance therapeutic window and understand non-genetic mechanisms driving escape from treatment in mesothelioma. To summarise these multi-layered findings, the integrated genetic, transcriptomic, and bioenergetic profiles of our mesothelioma PDO cohort are summarised in Table 1.

Our findings indicate that the route to cisplatin tolerance is not uniform, and it is contributed to by the tumour’s underlying mutational profile. By maintaining key mutations, with the relevant imprinting-germline versus acquired-drivers of the original tumour, such as the BAP1-loss signature seen in PDO3 and PDO4 or the MTAP-loss/KRAS-mutant profile of PDO1, these preliminary models lead to the hypothesis that certain genetic alterations may create a dominant, stable convergent baseline that remains refractory to global transcriptomic remodelling during chemotherapy exposure. Conversely, in the absence of these functional resistance drivers, tolerance is achieved through significant metabolic plasticity. The biological diversity captured across our cohort provides a robust co-clinical framework, where the distinct transcriptomic and metabolic dependencies of each PDO model mirror the aggressive clinical reality of the source malignancy.

Our data suggest that PDO1 serves as an individual case study of extreme adaptive plasticity during the emergence of a cisplatin-tolerant phenotype. We do not propose that PDO1 represents the entire BAP1-retained population. The concurrent oncogenic KRAS mutation likely acts as an independent driver for the mobilisation of bioenergetic resources observed in this model. Clinically, this patient presented with a highly aggressive, poorly differentiated epithelioid tumour, with one-month survival post-diagnosis at the age of 47. Despite having MTAP retained, typically associated with a more favourable mesothelioma prognosis [35], the clinical course was likely exacerbated by a concurrent NSCLC diagnosis harboring an oncogenic KRAS (G12D) mutation and driven by a global mobilisation of bioenergetic resources that correlates with a simultaneous increase in mitochondrial respiration and glycolytic flux alongside the upregulation of *VPS52*, *SYNGR3*, and *PROM2*. Our data have shown a significant increase in bioenergetic flux in PDO1, characterised by a 2.8- to 3.1-fold simultaneous increase in both mitochondrial respiration and glycolysis, suggesting global mobilisation of resources to fuel the non-genetic survival mechanisms, such as epigenetic remodelling and drug efflux [14]. However, without maximal respiration data to calculate spare respiratory capacity, the full extent of this metabolic plasticity remains to be quantified. Clinically, in lung cancer, cisplatin resistance in *KRAS*-mutants is acquired by activating ERK/JNK signaling, which inhibits AlkB homolog 5 (ALKBH5) N6-methyladenosine (m6A) demethylase activity by regulating posttranslational modifications (PTMs) of ALKBH5 [36]. Consequently, the KRAS mutant leads to a global increase in m6A methylation of mRNAs, particularly damage-specific DNA-binding protein 2 (DDB2) and XPC, which are essential for nucleotide excision repair. This methylation stabilises the mRNA of these two genes, thus enhancing NSCLC cell capability to repair platinum-induced DNA damage and avoid apoptosis, thereby contributing to drug resistance [36]. The upregulation of *PROM2* points towards an adaptive strategy that may manage iron homeostasis and prevent ferroptosis during the stressful transition to drug tolerance [30], while the concurrent upregulation of *VPS52* further points towards an enhanced endosomal and extracellular vesicle (EV) trafficking network, which we hypothesise may be used to sequester or export cisplatin to maintain cellular viability [31]. However, we emphasise that this is currently a correlative observation, with future functional studies utilising gene knockdown required to prove if this network actively sequesters cisplatin. While our transcriptomic profiling highlights a strong association between the upregulation of vesicle mobilisation genes (such as *SYNGR3*, *VPS52*, and *PROM2*) and the emergence of the cisplatin-tolerant state, we acknowledge that this proof-of-concept study relies on observational ex vivo data. Functional validation of these target genes is required to confirm whether disrupting this vesicular transport network can therapeutically dismantle the cisplatin-tolerant state and re-sensitise mesothelioma cells to platinum-based therapies.

Collectively, these findings suggest that for tumours like PDO1, chemoresistance is not a passive trait but a high-energy adaptive response that may enable a sub-population of cells to function as a reservoir for recurrent, refractory disease.

The clinical fidelity of our platform is further shown by PDO2, which exhibited an inherent, de novo resistance profile in its treatment-naive state, resulting in a negligible IC50 shift (1.9-fold) following cisplatin treatment. This minimal separation suggests that the PDO successfully preserved the aggressive, pre-programmed resistance of the parent tumour, which clinically presented as a highly aggressive disease with one month survival post-diagnosis. The patient’s rare presentation of concurrent epithelioid mesothelioma and acral lentiginous melanoma (ALM) with pelvic nodal metastasis, and prior prostate cancer, and in the absence of indicators of germline mutation (BAP1 retained), underscores a state of extreme systemic malignancy. Metabolically, PDO2 reflected this aggression through a hypermetabolic hybrid phenotype, characterised by the simultaneous increase in mitochondrial respiration and glycolysis. This global use of bioenergetic resources provides a robust metabolic engine to fuel rapid proliferation and survive therapeutic stress [14]. At the molecular level, the cisplatin-treated PDO2 displayed upregulation of *VPS52* and *SYNGR3*, alongside the downregulation of *PROM2*. The overexpression of *VPS52*, a component of the Golgi-associated retrograde protein (GARP) complex and possibly involved in sorting between endosomes and the trans Golgi network, may play a role in cisplatin trafficking away from the nucleus [37]. Similarly, the upregulation of *SYNGR3* and loss of *PROM2* are increasingly recognised as markers of altered extracellular vesicle (EV) dynamics and ferroptosis resistance, respectively, suggesting that while PDO2 is bioenergetically fixed in a resistant state, it may harbor unique vulnerabilities in its membrane trafficking and ferroptotic pathways [30,38].

The high-fidelity representation of the parent tumour in our study is further validated by PDO3 and PDO4, which faithfully recapitulate the BAP1-deficient/MTAP-retained molecular subtype of mesothelioma. Specifically, we observed a simultaneous reduction in mitochondrial respiration, with basal OCR decreasing by 12% in PDO3 and 31% in PDO4, alongside a concurrent suppression of glycolytic flux (ECAR) by 12% and 14%, respectively. This shift into a hypometabolic energy phenotype suggests that these cells have entered a persister state that provides a survival advantage by limiting replication-dependent cytotoxicity and minimising the production of endogenous reactive oxygen species to raise the threshold for oxidative stress-induced apoptosis [39]. While this strategy mirrors the hallmark behaviour of the DTP-like state, with exit from the cell cycle to weather therapeutic stress, the dual suppression also indicates a state of metabolic inflexibility [39]. Transcriptomic analysis confirmed this inflexibility, as the tolerant state was marked solely by the upregulation of *SYNGR3*, without significant alterations in *VPS52* or *PROM2* seen in more plastic models. The persistence of this intermediate biological reservoir through reversible phenotypic plasticity underscores the primary barrier to long-term survival: the eventual development of recurrent and refractory disease once these dormant sub-populations re-enter the cell cycle constitutes a major cause of treatment failure [6].

The PCA results underscore the significant molecular diversity inherent in mesothelioma, even when modelled ex vivo. The clear segregation of models based on BAP1 and MTAP status, two clinically relevant markers in mesothelioma, suggests that PDOs are faithful representatives of their original tumours. The proximity of PDO3 and PDO4 suggests that BAP1 loss creates a dominant molecular phenotype that overrides individual patient backgrounds, potentially identifying a specific cluster of mesothelioma that may respond similarly to targeted therapies such as PARP inhibitors, HDAC inhibitors, or immune checkpoint blockade [40]. This indicates that BAP1-deficient tumours occupy a stable baseline that remains largely refractory to global transcriptomic remodelling during chemotherapy exposure.

In contrast, the isolation of PDO2 on the PC2 axis likely reflects the unique metabolic and signalling impact of its MTAP loss and KRAS mutation, a combination known to drive aggressive tumour biology and altered nucleotide metabolism. This molecular aggression aligns with the patient’s complex clinical reality, characterised by extreme systemic malignancy, including concurrent ALM and prostate cancer. The tight clustering of treatment-naive and cisplatin-tolerant pairs in PDO2/3/4 suggests that the mechanisms of cisplatin tolerance in these backgrounds are driven by subtle, hard-wired cellular programs or epigenetic modifications rather than a complete overhaul of the transcriptome. However, the wider spatial separation observed in PDO1 suggests that patients with retained BAP1/MTAP status might undergo more plastic molecular remodelling during treatment.

We acknowledge that the primary limitation of this study is the small cohort size (*n* = 4 PDO models); the profound inter-patient heterogeneity observed across this small sample set provides compelling evidence that the routes to cisplatin tolerance are highly divergent.

The establishment of primary PDOs from pleural effusions, followed by the long-term generation of time-matched, cisplatin-tolerant pairs, is a highly time- and resource-intensive process [19]. Consequently, this study was designed as a proof-of-concept investigation to validate the utility of this 3D platform in capturing the highly transient cisplatin tolerance state. While this sample size is sufficient to demonstrate that therapeutic escape pathways are divergent and can be tracked using our multi-omics pipeline, larger subsequent studies are required to assess the broader reproducibility of these specific metabolic and transcriptomic dependencies.

The sample size used in this study aligns with the established literature by exceeding the published median for treatment-specific PDO cohorts [41], while also accounting for the significant technical challenges of 3D metabolic phenotyping [42]. Ultimately, this focused proof-of-concept fulfils the field’s immediate need to establish rigorous, standardised baseline protocols before pursuing large-scale clinical expansion [43].

As this study was designed strictly as a proof-of-concept methodological investigation, our findings, including the association between BAP1 status and metabolic phenotypes, should be interpreted as preliminary observations rather than generalisable rules. While this small cohort successfully demonstrates the platform’s utility in capturing the cisplatin-tolerant state, definitively confirming universal resistance mechanisms requires further validation. Testing these dependencies in larger, clinically well-characterised cohorts and performing functional target validation are critical next steps planned for future studies.

The lack of a BAP1 and MTAP-retained KRAS wildtype organoid is also a limitation. Additional PDOs are required, and future CRISPR Knockout/in may also help to apportion the relative effects.

Future research will aim to correlate these adaptive resistance mechanisms not only with foundational genetic profiles (such as BAP1 and MTAP) but also with the patient’s specific clinical stage of progression at the time of effusion collection. Serial samples will also be tested. Another limitation of the current study is the absence of longitudinal ‘drug holiday’ experiments. Because we did not formally quantify the reversibility of the IC50 shift following the removal of cisplatin, our models are most accurately defined as a cisplatin-tolerant phenotype. However, this reflects the clinical situation where alternative therapy is often started immediately once one therapy fails. However, future studies utilising drug holidays are planned to confirm the reversibility of these metabolic and transcriptomic adaptations.

Ultimately, the use of a high-fidelity 3D organoid platform ensures our findings are biologically representative of the complex in vivo tumour environment, bypassing the artificially heightened cisplatin sensitivity demonstrated in standard 2D cultures [44,45].

## 5. Conclusions

The capacity to predict therapeutic trajectories in pleural mesothelioma is frequently limited by the inability of traditional preclinical models to capture the complex genomic context of the patient. While engineered cell lines with targeted deletions provide mechanistic insights, they often fail to recapitulate the nuanced biological reality of the original tumour. The clinical response to chemotherapy in BAP1-deficient patients can vary significantly depending on whether the loss is germline or acquired. Similarly, while MTAP loss is a surrogate for CDKN2A deletion, these events are not functionally identical; the preservation of the genetic reality of the parent tumour in the PDO model may allow for the interrogation of MTAP-specific potential metabolic dependencies within their authentic genetic context.

While the current study focuses exclusively on cisplatin tolerance, expanding this 3D modelling approach to include broader therapeutic panels is an important next step. Currently serving as a hypothesis-generating tool, this platform may eventually help to differentiate between patients requiring consistent therapeutic strategies versus those needing adaptive, multi-line approaches to account for treatment-induced molecular shifts. By faithfully recapitulating the dynamic transition from drug sensitivity to this intermediate tolerant state, these PDO models provide a unique co-clinical window to identify non-genetic metabolic dependencies that are typically lost in traditional static models, thereby facilitating the discovery of therapeutic strategies to eradicate the reservoir of recurrent disease. Ultimately, this framework serves as a universal model for understanding therapy-induced plasticity that may allow us to stratify patients and develop personalised, combination-based interventions to eradicate refractory disease across a variety of aggressive malignancies.

## Figures and Tables

**Table 1:** Integrative summary of genomic, transcriptomic, and metabolic profiles in mesothelioma PDOs.

PDO ID	Age at Diagnosis (Years)	Sex	Survival (Months)	BAP1 Status	MTAP Status	KRAS Status	Metabolic Phenotype	VPS52	SYNGR3	PROM2
PDO 1	47	Male	1	+	+	+	Hypermetabolic baseline/hybrid state. Concurrent high increase in bioenergetic flux.	↑	↑	↑
PDO2	77	Male	1	+	−	N/A	Hypermetabolic baseline/hybrid state. Concurrent increase in bioenergetic flux.	↑	↑	↓
PDO3	74	Male	20	−	+	N/A	High reliance on mitochondrial respiration; predicted convergent stable baseline.	↓	↑	↑
PDO4	80	Female	5	−	+	N/A		↔	↑	↔

## Data Availability

All data are included in the paper or attached as Supplementary Materials.

## Supplementary Materials

The following supporting information can be downloaded at https://www.mdpi.com/article/10.3390/cancers18101500/s1, Table S1: Assessment of IC50 fold changes during the establishment of patient-derived organoid models; Table S2: Transcriptomic profiling of differentially expressed genes in matched cisplatin-naive and cisplatin-tolerant patient-derived organoid models.

## Abbreviations

The following abbreviations are used in this manuscript:

ACK	Ammonium–Chloride–Potassium
AdVDMEM/F12	Advanced Dulbecco’s Modified Eagle Medium/Ham’s/F-12
ALKBH5	AlkB homolog 5
ALM	Acral lentiginous melanoma
DDB2	Damage-specific DNA-binding protein 2
DTP	Drug-tolerant persister
ECAR	Extracellular acidification rate
EV	Extracellular vesicle
FCCP	Carbonyl cyanide-p-trifluoromethoxyphenylhydrazone
GARP	Golgi-associated retrograde protein
IC50	Half-maximal inhibitory concentration
IHC	Immunohistochemistry
m6A	N6-methyladenosine
NATA	National Association of Testing Authorities
NSCLC	Non-small cell lung cancer
OCR	Oxygen consumption rate
PCA	Principal Component Analysis
PDO	Patient-derived organoid
PM	Pleural mesothelioma
PRMT5	Protein Arginine Methyltransferase 5
PTMs	Posttranslational modifications
RCPA	Royal College of Pathologists of Australia
RI	Resistance Index
SD	Standard deviation
SEM	Standard error of the mean
TEM	Transmission electron microscopy
WT	Wild type
