# Supplementary material for: A High-Fidelity Patient-Derived Organoid Platform Recapitulates the Dynamic Metabolic Landscape of Cisplatin Tolerance in Mesothelioma
Source: Cancers (Basel). 2026 May 7;18(10):1500. doi: 10.3390/cancers18101500 (PMC13204214; doi:10.3390/cancers18101500)
Supplement: Supplementary file 1 [file cancers-18-01500-s001.zip › cancers-4253702-supplementary.pdf]

Supplementary Materials

A High-Fidelity Patient-Derived Organoid Platform Recapitulates the Dynamic Metabolic Landscape of Cisplatin Tolerance in Mesothelioma

Zivile Useckaite [1,*,†], Ashleigh J. Hocking [1,†], Lauren A. Mortimer [1], John Salamon [2], Simon Lee [2], Yazad Irani [1], Lucy Franzon [1], Arya L. Arul [1], Sarita Prabhakaran [1] and Sonja Klebe [1,3]

[1] Flinders Health and Medical Research Institute, College of Medicine and Public Health, Flinders University, Adelaide, SA 5042, Australia
[2] South Australian Genomics Centre, SAHMRI (South Australian Health and Medical Research Institute), North Terrace, Adelaide, SA 5000, Australia
[3] Department of Surgical Pathology, SA Pathology at Flinders Medical Centre, Adelaide, SA 5042, Australia
* Correspondence: zivile.useckaite@flinders.edu.au
† These authors contributed equally to this work.

Table S1. Assessment of IC50 fold changes during the establishment of patient-derived organoid models.

**PDO1**	**IC50 P5 (T0)**	**IC50 P15**	**RI**
	1.47	3.3	2.24
**PDO2**	**IC50 P5 (T0)**	**IC50 P11**	**RI**
	1.25	1.75	1.4
**PDO3**	**IC50 P6 (T0)**	**IC50 P15**	**RI**
	1.5	4.7	3.13
**PDO4**	**IC50 P5 (T0)**	**IC50 P14**	**RI**
	0.76	3.2	4.2

PDO = Patient Derived Organoid
RI = Resistance Index
P = Passage
T = Time

Table S2. Transcriptomic profiling of differentially expressed genes in matched cisplatin-naive and cisplatin-tolerant patient-derived organoid models.

**gene_id**	**PDO1_cis**	**PDO1_cont**	**PDO2_cis**	**PDO2_cont**	**PDO3_cis**	**PDO3_cont**	**PDO4_cis**	**PDO4_cont**
ACP7	5.9617	3.2157	7.9566	6.065	4.785	3.2157	5.2201	3.9491
ADAMTS15	9.0639	10.4625	10.22	13.2728	7.0527	8.2496	8.4565	9.3252
AGER_3	3.2157	5.0219	3.2157	8.5882	7.6815	7.5942	3.2157	8.4774
AGT	9.6813	6.1188	5.828	4.4671	4.9881	4.1201	4.2596	3.6886
AK6	3.2157	8.989	3.2157	3.2157	3.2157	9.9998	10.6747	3.2157
ALOX5AP	9.4527	10.8142	4.5871	6.8148	4.0231	4.5486	4.727	5.7262
ANKRD1	6.8326	5.3642	14.6493	9.1396	10.782	7.9504	13.5288	12.4941
ANKRD36_1	9.0205	11.7896	10.7775	11.5369	8.7222	12.1053	9.035	12.073
APLN	3.6625	5.6099	9.5559	11.842	8.8392	11.5494	8.9223	9.4502
ARHGAP11B	7.8091	10.5495	3.2157	8.1276	3.2157	7.5403	8.3116	9.8098
ATF3	11.5129	9.862	12.1327	11.1272	10.7977	8.5964	10.5484	9.2578
ATL1	9.9395	5.3754	11.0431	10.8539	6.367	4.4186	6.7833	4.0422

ATP23	4.2027	7.4112	3.2157	8.6384	7.1445	8.0054	3.2157	6.8648
BCAS1	10.5843	5.3395	4.2986	3.2157	3.8671	3.2157	4.5026	3.7059
C10orf67	5.1151	6.2416	7.3228	8.8561	4.1163	5.0934	4.0474	6.786
C16orf46	3.2157	7.5347	3.707	8.2227	7.2672	7.7559	3.2157	7.0184
C1QTNF2	6.9428	4.25	8.1215	6.7746	5.052	4.2283	6.873	4.3784
C6orf136_5	3.2157	7.0996	3.2157	6.2982	5.7833	3.2157	3.2157	7.5759
CA12	11.1484	14.4804	12.3739	15.9675	14.7669	16.2035	8.9037	9.1616
CA9	6.7162	12.5966	5.1465	13.2498	9.0172	13.5382	4.9017	5.7583
CACNB4	7.3511	6.0463	6.6759	5.1751	4.841	4.2096	7.9502	5.5536
CALB2	9.7092	10.3655	6.9021	12.1959	8.6745	11.863	3.2157	6.5918
CCDC148	6.9871	5.0658	8.3421	7.6976	6.683	5.127	6.5908	4.6591
CCDC162P	7.4005	5.0608	4.2644	3.7398	5.2537	4.4756	5.6867	4.4379
CCL5	9.6813	3.2157	6.3744	3.2157	11.863	11.6673	12.0689	3.2157
CCN3	12.7764	10.867	12.887	10.5929	12.0851	10.2825	10.181	9.6172
CD70	9.944	6.1774	9.5286	5.6669	11.1707	7.695	11.711	11.192
CDON	9.3095	10.3059	11.9415	13.6636	10.3457	12.2011	11.3265	12.3253
CEACAM1	15.1327	11.0491	7.6008	6.8865	8.5888	6.2145	5.4185	3.9601
CEMIP	8.1024	9.7183	15.3565	18.2865	15.3678	17.3699	13.3871	14.5091
CEND1	13.4557	6.784	7.1063	5.0002	4.0217	3.2157	7.1985	5.1382
CHCHD10	13.2982	12.4817	9.9587	3.2157	6.8319	4.7757	10.7054	3.907
CHCHD10_1	9.3176	12.2496	3.2157	8.8874	3.2157	4.7844	3.2157	8.4039
CHRM2	3.6913	3.2157	9.6667	6.207	4.1537	3.2157	3.7229	3.2157
CMPK2	11.5283	6.7539	5.2746	4.7652	12.9279	11.2396	11.9712	9.7109
CNTF	3.2157	4.5962	5.1954	7.7951	4.2492	5.5547	6.0018	6.9521
COLEC10	6.9631	6.0439	13.5295	12.2272	10.2796	6.3536	10.6949	7.5491
CST7	4.6077	3.2157	6.5043	4.8723	6.4261	5.1684	7.8925	6.6642
CTSS	12.908	9.7706	10.3935	7.5249	5.5065	5.0417	6.1128	4.6588
CUTA	12.743	10.7147	13.541	12.4614	12.6509	11.5022	13.1166	11.0852
CYLD-AS1	6.3223	5.7199	8.2924	6.6543	6.5827	5.4217	6.7703	5.7052
DACH1	7.0398	10.5283	5.4188	6.5957	3.2157	3.2157	6.5196	8.4185
DDX3ILA1	9.6549	7.3395	4.7909	4.2061	6.0398	4.7611	4.277	4.0411
DEPTOR	11.6418	9.6649	9.4936	6.655	12.4518	11.5452	9.1608	6.6345
DGKG	9.9218	7.9438	9.4142	8.4043	10.1021	8.9042	8.7646	7.0904
DHX16_2	3.2157	10.8436	3.2157	7.8899	3.2157	8.5802	10.0164	11.345
DHX16_4	3.2157	3.2157	9.9768	3.2157	3.2157	10.7548	3.2157	10.5822
DSP	14.7316	14.416	14.2965	12.2018	6.0221	4.5357	12.0942	8.7595
E2F2_1	10.8441	3.2157	5.9741	3.2157	9.285	3.2157	10.2403	9.9384
EDN1	10.0499	6.0763	11.0892	9.1433	5.1715	4.4278	5.8334	4.8787
EGLN3	7.7221	11.521	6.1981	12.5361	5.8745	7.8736	5.8488	6.5723
ERRFI1	15.8648	17.3007	12.2268	14.6157	11.5812	12.69	13.974	14.953
FAM149A	8.4431	6.6937	4.1669	3.2157	6.6863	4.8189	3.7694	3.2157
FAM157A_1	6.717	3.2157	5.4699	3.2157	5.2129	3.2157	4.9571	3.2157
FAR2	10.3136	9.4099	9.6438	8.8586	8.1028	6.7661	7.0238	5.8654
FOLR3	3.2157	3.2157	10.0838	8.5135	7.9421	5.7212	7.2035	5.2254
FOXP1-DT	7.3818	8.8693	5.8817	6.96	6.6158	7.5895	4.5942	6.358
FREM2	12.4038	9.142	4.9119	3.2157	3.2157	3.2157	5.529	3.8151
GABBR1_5	3.2157	3.2157	7.8846	3.2157	3.2157	12.1146	3.2157	8.6132
GABRP	15.9662	12.1493	3.6989	3.2157	3.2157	3.2157	3.8941	3.2157
GAD1	6.3	5.536	7.8469	6.0139	6.8922	5.0618	6.2828	5.4938

GALNT5	10.5258	8.1193	11.7834	10.1426	4.3455	3.2157	4.8091	4.0934
GATA5	3.6635	3.9912	4.7925	7.9217	10.3485	11.2264	7.3872	10.5465
GATA6	10.8975	13.2086	12.9765	14.8235	13.0039	13.7547	12.7656	13.8176
GBP4	10.9635	8.6467	6.5153	4.871	10.3218	8.1609	12.7099	10.0116
GDF6	4.8609	3.2157	12.5698	9.3371	4.5059	3.2157	6.2367	5.1858
GJA1	11.0909	13.1566	12.7894	13.9989	13.0948	15.0895	13.5656	14.2756
GLRX	13.6398	11.9055	13.0707	12.4104	8.8927	7.5602	10.335	9.2887
GPAT3	12.0228	11.2494	7.0053	5.5788	8.36	6.7461	8.6003	6.9867
GTF2H4_5	9.7033	3.2157	3.2157	11.1582	9.7005	3.2157	10.1522	3.2157
H2BC14	4.5092	5.2252	5.9439	6.9196	11.3749	12.5135	12.5294	13.6971
HAPLN1	9.2539	13.5293	5.5371	8.8786	3.2157	3.8605	3.2157	6.5456
HCG11	3.2157	9.7896	3.2157	9.4179	3.2157	3.2157	9.7227	3.2157
HCLS1	8.5682	5.9288	4.5465	3.7506	4.8124	3.6828	7.0544	5.1194
HECW2	10.2312	9.563	11.0622	8.0776	9.754	7.408	10.5114	9.2105
HERC2P9	9.6258	3.2157	10.1544	3.2157	3.2157	10.2085	10.1928	3.2157
HERC6	13.4849	9.4166	9.5528	7.2198	13.8102	11.4815	12.8794	10.5198
HKDC1	10.1149	8.4751	11.3127	9.237	9.405	7.7895	8.3941	7.5793
HRK	9.9028	6.2479	9.4645	7.4448	6.1282	4.7935	5.057	4.7855
ID3	7.9757	3.2157	8.8241	3.2157	10.289	3.2157	8.6827	9.4816
IDO1	13.7621	9.3272	5.6261	4.8013	3.2157	3.2157	6.1742	4.2546
IFI30	11.9626	9.2408	9.6064	8.3924	10.448	8.5231	9.5035	7.2743
IFI35	10.566	9.4015	11.3389	10.4879	12.7716	11.3707	12.8532	11.3086
IFI44	12.5265	11.2533	9.0961	8.0517	13.6234	11.9595	14.0834	11.6158
IFI6	14.5036	12.4557	11.1328	10.4898	15.4418	13.4743	16.0766	12.9096
IFIH1	13.1467	10.6808	11.9347	10.9503	13.4825	12.2012	13.7315	11.864
IFIT1	11.97	10.1652	13.4495	12.36	14.1892	11.5336	14.6863	13.0097
IFIT2	12.9587	10.8852	13.6559	11.1684	13.3676	11.4595	14.1533	12.5662
IFIT3	12.9365	10.4551	14.0502	12.1917	14.3728	11.9374	14.4868	12.4685
IFIT5	11.523	9.8607	12.5095	11.4832	13.1775	12.0421	13.5053	12.2317
IFNGR2_1	5.4333	6.6536	5.2796	6.3217	5.5457	6.2172	6.1182	7.6231
IL23A	7.8288	5.1834	5.4526	4.1144	4.9373	4.5464	5.8474	4.4313
ISG15	13.7704	11.2207	10.7638	9.4828	14.0023	11.096	14.1144	11.8647
KCNQ1	6.1233	9.7024	3.2157	5.1741	3.2157	3.2157	3.2157	5.4216
KCTD11	3.2157	9.3169	9.9267	3.2157	3.2157	3.2157	10.3169	3.2157
KIT	6.6179	3.2157	9.5825	5.1708	3.2157	3.2157	7.5313	4.8424
KLHDC7B	14.0114	12.6588	6.1427	4.9997	9.2047	7.4795	7.7035	5.309
KRT17	17.0014	14.7954	7.1782	4.8716	8.3079	7.4162	7.2182	5.8647
KYNU	14.9458	13.8762	5.7823	3.9723	5.3957	3.2157	5.0083	4.0012
LDB2	6.0713	8.6425	3.7166	10.6199	5.0025	8.5268	5.0013	12.2808
LENG9_1	9.5953	3.2157	3.2157	3.2157	3.2157	9.6742	9.0741	3.2157
LINC00957	5.1641	6.1214	6.6268	8.2904	5.4589	7.036	5.1778	5.7445
LINC00973	10.6339	8.8073	9.8279	7.2266	11.1719	8.9474	10.9179	7.8612
LINC02051	4.584	3.2157	6.7349	5.17	7.1078	5.0851	6.2547	3.2157
LINC02984	12.777	10.0012	5.4344	4.4668	7.3216	4.8991	6.2174	4.7242
LIPH	12.1091	8.6934	10.4906	7.6015	6.4224	3.9997	5.3092	4.16
LOC100132154	8.545	4.9366	4.8433	3.2157	4.4142	3.8126	5.6413	4.3503
LOC100294145_2	3.2157	6.6638	10.0918	3.2157	10.1495	3.2157	3.2157	3.2157
LOC100310756	3.2157	6.9998	3.2157	4.8034	3.2157	9.4912	8.1941	8.1797
LOC100506253	6.5136	4.6563	4.0331	3.2157	5.4726	4.0019	4.8475	3.2157

LOC105372401	5.677	7.3289	9.6254	11.5102	9.5655	10.2131	9.6328	10.6379
LOC105373596	3.2157	4.5953	4.5258	9.0891	3.2157	3.6738	5.1936	8.4801
LOC105374002	6.6338	5.3779	6.1399	4.7865	6.4013	5.6453	6.6185	5.2404
LOC105376292	10.4959	7.2697	3.2157	3.2157	5.5434	4.486	4.6835	3.2157
LOC105378532	5.5215	4.1876	6.7292	5.2892	5.8643	4.2778	4.635	4.0856
LOC107985869	5.042	3.2157	11.0595	8.0692	6.1692	4.4756	8.356	7.0005
LOC107986396_1	8.3291	4.8757	4.1568	3.2157	4.0217	3.2157	3.6909	3.2157
LOC124900812	4.3312	4.8388	4.7775	7.0418	5.4386	6.9031	4.6784	5.7227
LOC124904260	3.2157	5.144	4.7974	6.5625	3.2157	6.0935	3.2157	5.873
LOC124909489	10.2454	9.298	14.4939	12.126	6.4672	5.5222	4.9594	4.4767
LOC339685	6.1318	8.822	3.2157	4.999	3.685	4.8984	3.2157	4.2562
LOC728554	3.2157	7.1931	3.2157	10.5596	3.2157	9.2432	6.7996	10.2499
LOC728554_2	7.2326	3.2157	10.4898	3.2157	9.0509	3.2157	10.4623	9.9219
LRP6	10.768	12.772	11.7387	12.9858	9.4645	9.9467	7.7341	10.2248
LSM2	3.2157	10.9259	3.2157	11.6073	3.2157	10.9492	3.2157	10.5735
LSM2_5	3.2157	3.2157	10.0064	3.2157	10.7059	3.2157	10.9763	8.6573
LTC4S_1	7.5467	8.467	4.7738	3.2157	7.8612	3.2157	8.2432	3.2157
LURAP1L	11.5678	10.4678	9.421	7.0273	9.7264	6.9364	9.4827	8.631
LY6S_1	9.6508	5.8872	3.2157	3.2157	4.5093	3.2157	4.2628	3.2157
MAB21L3	8.6031	7.4426	5.479	4.6529	6.6193	5.573	6.1867	4.5968
MACROH2A2	10.4375	8.7363	9.5941	8.8133	8.1507	5.774	7.4301	6.1224
MAP2	13.2875	8.0202	11.5837	7.4747	9.0767	8.2007	10.1998	7.5857
MCPH1-DT	8.3103	3.2157	8.2125	3.2157	7.3645	7.4315	7.8482	3.2157
MICA_1	3.2157	8.4899	9.145	3.2157	10.4442	3.2157	6.8411	3.2157
MIR210HG_1	7.1082	9.627	6.3537	9.4796	6.358	8.7842	7.9944	7.9478
MKX	4.4602	3.9998	7.7011	4.9555	3.8847	3.2157	5.959	4.038
MROH6	11.2002	5.267	9.4781	10.2098	8.6514	3.2157	8.7866	3.2157
MTMR10_2	8.7779	3.2157	11.6627	3.2157	3.2157	9.3892	10.3258	3.2157
MTSS1	7.9294	9.2056	13.3866	14.6524	14.1249	14.7523	13.8728	14.9833
MUC6_2	3.2157	13.5781	3.2157	3.2157	3.2157	3.2157	3.2157	3.2157
MX1	14.0724	11.0252	10.8985	10.5644	16.1319	14.3204	15.7359	13.0634
NDUFA6	8.2573	10.6665	9.401	11.5218	3.2157	3.2157	3.2157	3.2157
NEB	7.7296	5.9501	5.8031	4.8754	5.5098	4.6249	8.7405	7.4712
NEURL3	8.9273	5.8524	6.7434	5.2973	6.3579	5.1506	7.2942	4.5293
NGFR	8.922	7.0172	6.6071	5.9197	11.0338	8.3098	15.4083	10.4554
NKRF	4.9554	7.5417	3.2157	6.1157	3.2157	6.9697	6.7304	7.4297
NOTCH2NLR	3.2157	7.2391	7.9191	7.5487	3.2157	7.038	3.2157	7.3073
NOTCH2NLR_1	7.8202	3.2157	3.2157	3.2157	7.2507	3.2157	7.5926	3.2157
NRK	4.7564	3.6675	13.7471	10.7123	4.7144	3.8618	8.2687	4.0308
OAS2	13.5375	12.4393	11.3282	10.2083	15.1963	12.9684	14.3845	11.765
OASL	12.2427	10.8148	10.0962	6.5749	12.3258	10.3922	12.5107	10.8846
PARP9	13.9229	11.2052	12.6049	12.2088	14.2269	12.9319	14.6229	12.5531
PBX1	13.332	14.2171	13.7366	14.7038	14.6945	16.1416	11.282	13.8273
PCDH12	8.9679	6.3495	4.5256	3.2157	3.8767	3.2157	4.5948	3.2157
PCSK9	12.6676	8.5405	9.1327	6.6756	3.2157	3.2157	10.7943	9.2636
PDE1C	5.5455	4.6282	14.9457	13.9412	4.0956	3.6836	8.7324	7.1718
PDGFRA	8.5965	11.8872	9.3537	11.7574	13.333	14.9036	8.6187	11.3505
PDK1-AS1	6.0106	8.0073	7.0054	8.2579	5.5206	6.9324	5.5138	6.5378
PLAAT2	8.7672	6.2984	3.8905	3.2157	4.5296	3.6822	5.5141	4.5298

PLAAT4	12.4756	9.6942	9.674	8.9877	9.2199	8.1628	9.7192	7.9099
PLEKHS1	13.4254	11.6499	9.8677	6.3347	7.8097	5.1852	7.6703	5.0493
PLLP	7.5521	10.7939	7.9807	11.9661	7.9793	8.8822	6.6424	7.8955
POPDC3	3.2157	3.2157	10.2316	7.2511	10.3901	8.4741	9.8691	7.0153
PPM1K	10.7421	9.4971	8.2917	5.8308	9.5571	7.7787	9.4548	8.4569
PPP1R3G	5.2031	6.3823	7.2396	8.0957	7.7122	9.3967	6.7316	8.2435
PPP2R3B_1	7.8117	6.5528	3.2157	5.587	3.2157	9.4821	6.4296	10.8183
PRDM1	12.0693	10.8655	6.3367	5.1657	9.2483	7.3937	5.6716	4.3837
PRICKLE2	10.121	12.3187	12.5755	12.9161	7.6994	10.29	9.4336	10.9279
PRKG1	5.958	4.4718	11.228	8.6351	9.5918	8.8262	5.3828	4.0892
PROM2	14.7777	3.2157	4.7326	8.9481	5.2048	3.2157	3.2157	3.2157
PRPF31	11.7988	3.2157	3.2157	12.5309	3.2157	3.2157	3.2157	9.2844
PSG1	4.4567	3.2157	15.5797	12.7341	6.9357	5.657	9.4024	7.1434
PSG2	3.2157	3.2157	8.4072	6.2474	5.2164	4.0036	6.441	4.7841
PSG9	4.1063	3.2157	10.2039	7.6197	4.6984	4.2488	7.2377	5.7859
PSMB8_6	10.8321	3.2157	3.2157	3.2157	10.3364	3.2157	8.5361	10.9028
PTGS1	7.355	8.5123	5.3595	7.7967	6.6618	8.9122	5.0074	6.7753
RAET1L	12.1504	5.5798	4.0292	3.2157	4.021	3.2157	4.0297	3.2157
RAPGEF4	6.7831	8.1365	5.5788	7.0468	5.4133	6.9728	5.4497	5.8563
RASEF	12.2253	8.4026	6.5665	4.5618	10.7379	8.8904	8.9883	8.3708
RASGRP3	7.4816	5.9165	7.0321	5.6436	7.1152	5.8759	7.7313	6.2818
RCAN2	10.5988	3.2157	4.4674	3.7293	8.6574	8.1583	7.7679	3.2157
RIGI	12.9297	11.4868	12.046	11.375	13.7724	12.2836	13.5767	12.1577
RIPOR3	4.1994	4.2658	9.1189	10.422	7.3425	8.9889	4.9318	6.1591
RNA18SN3	17.3935	15.3789	17.3154	16.2506	19.3229	16.5854	16.6145	14.5665
RNF5_4	11.1589	3.2157	9.9202	3.2157	10.4914	3.2157	10.8835	3.2157
RPS9_3	11.1705	12.921	10.4819	12.6273	10.8673	12.1239	9.4796	11.4246
RRAGD	10.9263	6.5905	9.3928	7.8694	7.2383	5.3922	7.6605	6.0837
RSAD2	11.4832	7.086	6.9562	5.3665	12.9274	10.5713	13.4889	11.0606
SAMD9L	11.9746	9.2361	13.4372	12.6911	13.5283	11.7975	13.5795	11.0168
SCAND2P_1	7.0317	10.4603	7.9551	3.2157	8.4351	3.2157	9.6008	3.2157
SDHAP2_2	10.3236	3.2157	3.2157	9.8398	3.2157	3.2157	3.2157	10.2899
SEC14L6	3.2157	3.8581	8.9261	11.8139	6.3078	7.9058	7.0577	8.5408
SERTAD4_1	11.6243	6.3292	5.4867	3.2157	4.6715	4.4557	9.1706	5.5246
SFRP1	10.8455	5.8877	10.5306	9.7708	6.9666	5.7744	7.7922	4.0289
SLC16A10	7.4393	4.2711	3.5957	3.2157	3.9569	3.2157	6.6997	4.323
SLC16A6	9.5027	7.2447	7.1888	5.2325	8.3473	4.8016	4.575	3.6919
SLC1A1	10.6673	6.8146	11.0079	10.3729	6.9263	4.7338	6.5329	4.453
SLC26A9-AS1	9.9392	8.4793	4.0329	3.7399	7.4664	6.2822	5.7982	4.2555
SLC2A3	5.82	10.5221	14.1451	15.9732	8.0245	10.4584	11.9704	12.8392
SLC9A2	9.6517	5.9834	4.6247	3.7499	3.8895	3.2157	6.218	4.3727
SNAP25	3.2157	3.2157	6.6475	4.9427	8.2126	6.1509	8.3934	5.3874
SNORA79B	7.3469	3.2157	7.5393	3.2157	8.145	3.2157	7.6139	7.3066
SOWAHB	10.8265	6.2999	4.5989	3.2157	5.9741	3.8605	4.8485	4.8984
SPRR2D	13.0327	9.6897	4.2446	3.9522	3.875	3.2157	4.3478	3.2157
SPX	6.3565	4.9544	6.1123	3.9449	5.0696	3.2157	6.6319	3.9055
SRPX2	12.1295	10.4512	10.9821	9.4614	11.8368	11.2962	11.2681	8.7167
SSTR2	4.4429	3.9906	4.7964	3.2157	7.4667	5.3578	6.5227	3.8829
STK19	3.2157	3.2157	9.5306	3.2157	3.2157	9.0659	9.605	3.2157

STON1	7.8448	8.9495	12.0364	12.9098	10.5637	11.8589	10.6624	11.85
SUGCT-AS1	3.2157	3.2157	5.8305	4.1154	6.8502	5.0882	5.9053	4.5164
SYNGR3	8.4878	4.2985	6.7865	3.2157	7.7308	3.2157	7.5512	3.2157
SYNGR3_1	3.2157	4.2999	3.2157	9.0564	3.2157	6.9164	3.2157	7.0969
TAGAP	9.6318	3.8122	5.7103	4.2959	4.9464	3.2157	4.7008	3.2157
TBC1D3F	7.4604	3.2157	7.4412	8.4773	7.4402	3.2157	9.5132	3.2157
TCN1	14.9461	11.8033	3.8834	3.2157	6.2833	3.2157	4.3529	3.2157
TGM5	10.2814	5.6524	4.1254	3.2157	4.3785	3.2157	3.746	3.2157
THBS1	16.4698	13.8934	18.8536	18.0318	17.085	14.2848	19.6327	18.1714
THBS1-IT1	8.8245	6.2481	10.6704	9.8088	8.6157	6.2635	12.5771	10.7949
TIAM1	12.0441	10.8457	11.9807	10.4841	10.6541	9.2373	4.3248	4.2559
TLR1	12.4165	9.716	7.532	6.2206	8.2144	7.4649	7.0297	6.0021
TLR3	9.6229	6.8774	9.5261	8.7475	9.2525	7.2919	7.7658	5.3476
TMEM140	8.5503	7.4322	7.4337	6.2663	9.7396	8.3846	8.0831	5.4523
TMEM256	3.2157	9.4939	3.2157	10.4657	3.2157	10.3822	9.8244	3.2157
TMEM256_1	10.1653	3.2157	10.2239	3.2157	9.9817	3.2157	10.1344	10.9896
TNFSF18	4.2905	4.2991	9.9762	6.855	8.3263	6.7077	11.5994	7.2586
TPTEP2-CSNK1E	8.0599	9.1412	5.7775	6.6902	5.4733	6.7844	5.9767	7.0103
TRIM22	10.857	8.081	13.4232	12.5067	13.0885	10.9821	12.3957	9.6567
TSACC	7.3874	5.7669	6.6018	4.6323	5.9269	4.7813	5.3297	4.8464
TSEN34_9	5.04	3.2157	3.2157	11.5829	3.2157	7.8877	3.2157	3.2157
TTC5	3.2157	9.0401	3.2157	9.6872	10.7368	10.5002	3.2157	10.7688
UBL7	3.2157	11.2228	3.2157	3.2157	3.2157	11.2187	3.2157	11.5329
USP18	10.6794	6.4589	8.5195	7.4336	10.4419	8.4889	11.0936	8.6408
VGF	6.0167	4.927	8.903	7.3082	7.7197	6.4939	8.5901	7.315
VIT	4.6966	6.3423	9.6828	11.8873	4.0383	4.4395	4.3943	5.4148
VPS52	9.9791	3.2157	11.5252	3.2157	3.2157	10.2395	3.2157	3.2157
WHAMMP1_3	3.2157	3.2157	3.2157	10.1559	3.2157	8.3362	5.5291	3.2157
ZBP1	7.4967	4.3481	3.7705	3.2157	4.8953	3.8669	5.7324	4.5314
ZBTB22	10.1263	3.2157	3.2157	9.7977	3.2157	3.2157	11.1025	3.2157
ZNF185	11.2442	9.1204	10.7904	7.233	3.2157	3.2157	12.5484	8.714
ZNF365	10.3387	7.36	10.1591	8.7823	5.052	3.665	5.3559	3.6804
ZNF496-DT	8.335	10.1901	3.2157	7.1692	3.2157	10.2144	3.2157	9.8266

PDO = Patient derived organoid
PDO_cis = Patient derived organoid treated with cisplatin
PDO_con = Patient derived organoid untreated (time matched to PDO_cis)
